# The University of Zimbabwe College of Health Sciences (UZ-CHS) BIRTH COHORT study: rationale, design and methods

**DOI:** 10.1186/s12879-020-05432-6

**Published:** 2020-10-02

**Authors:** Kerina Duri, Felicity Z. Gumbo, Privilege T. Munjoma, Precious Chandiwana, Kudakwashe Mhandire, Asaph Ziruma, Andrew Macpherson, Simbarashe Rusakaniko, Exnevia Gomo, Benjamin Misselwitz, Lovemore Ronald Mazengera, the UZ-CHS Birth Cohort Team, M. Altfeld, M. Bunders, S. Rowland Jones, C. Dandara, V. Mleya, J. Mutambara, G. Kandawasvika, P. Kuona, S. Chimhuya, R. Nyamakura, S. Mtapuri-Zinyowera, S. P. Chandiwana, C. Marashiki, H. Mataramvura, E. Mazengera, N. Taremeredzwa

**Affiliations:** 1 Department of Immunology, University of Zimbabwe College of Health Sciences (UZ–CHS), P.O. Box A178 Avondale, Harare, Zimbabwe; 2 Department of Paediatrics and Child Health, UZ-CHS, Harare, Zimbabwe; 3 UZ-CHS Research Support Centre, UZ-CHS, Harare, Zimbabwe; 4 Department of Chemical Pathology, UZ-CHS, Harare, Zimbabwe; 5 Department of Obstetrics and Gynaecology, UZ-CHS, Harare, Zimbabwe; 6 Clinic for Visceral Surgery and Medicine, Inselspital Bern and Bern University, Bern, Switzerland; 7 Department of Community Medicine, UZ-CHS, Harare, Zimbabwe; 8 Department of Medical Laboratory Sciences, UZ-CHS, Harare, Zimbabwe

**Keywords:** Perinatal HIV/ART exposures, Breastfeeding, Maternal comorbidities, Immune-metabolic dysfunction, Microbiota dysbiosis, Adverse birth outcomes, Neonatal/infant/childhood adverse outcomes, Resource limited setting

## Abstract

**Background:**

Commencing lifelong antiretroviral therapy (ART) immediately following HIV diagnosis (Option B+), has greatly improved maternal-infant health. Thus, large and increasing numbers of HIV-infected women are on ART during pregnancy, a situation concurrently increasing numbers of HIV-exposed-uninfected (HEU) infants. Compared to their HIV-unexposed-uninfected (HUU) counterparts, HEU infants show higher rates of adverse birth outcomes, mortality, infectious/non-communicable diseases including impaired growth and neurocognitive development. There is an urgent need to understand the impact of HIV and early life ART exposures, immune-metabolic dysregulation, comorbidities and environmental confounders on adverse paediatric outcomes.

**Methods:**

Six hundred (600) HIV-infected and 600 HIV-uninfected pregnant women ≥20 weeks of gestation will be enrolled from four primary health centres in high density residential areas of Harare. Participants will be followed up as mother-infant-pairs at delivery, week(s) 1, 6, 10, 14, 24, 36, 48, 72 and 96 after birth. Clinical, socio-economic, nutritional and environmental data will be assessed for adverse birth outcomes, impaired growth, immune/neurodevelopment, vertical transmission of HIV, hepatitis-B/C viruses, cytomegalovirus and syphilis. Maternal urine, stool, plasma, cord blood, amniotic fluid, placenta and milk including infant plasma, dried blood spot and stool will be collected at enrolment and follow-up visits. The composite primary endpoint is stillbirth and infant mortality within the first two years of life in HEU versus HUU infants. Maternal mortality in HIV-infected versus -uninfected women is another primary outcome. Secondary endpoints include a range of maternal and infant outcomes. Sub-studies will address maternal stress and malnutrition, maternal-infant latent tuberculosis, *Helicobacter pylori* infections, immune-metabolomic dysregulation including gut, breast milk and amniotic fluid dysbiosis.

**Discussion:**

The University of Zimbabwe-College of Health-Sciences-Birth-Cohort study will provide a comprehensive assessment of risk factors and biomarkers for HEU infants’ adverse outcomes. This will ultimately help developing strategies to mitigate effects of maternal HIV, early-life ART exposures and comorbidities on infants’ mortality and morbidity.

**Trial registration:**

ClinicalTrial.gov Identifier: NCT04087239. Registered 12 September 2019.

## Background

Zimbabwe, a land-locked country in Southern Africa, is enduring a generalised human immunodeficiency virus type 1 (HIV-1) epidemic since 1985 [1]. With a population of 16 million, 1.3 million people were living with HIV-1 in 2016, of these, 200,000 were children under the age of 15 [2]. Like any other sub Saharan African (SSA) country, Zimbabwe is confronted with concomitant epidemics of HIV-1, co-infections [3–11] and non-communicable diseases (NCDs) such as cardiovascular diseases and metabolic syndromes [12–15]. Furthermore, SSA infants and their mothers are exposed to malnutrition [16] and poor access to clean water, sanitation and hygiene [17, 18].

The introduction of antiretroviral therapy (ART) in SSA has greatly improved maternal-infant health (2;19;20). In 2013, Zimbabwe adopted the Option B+ strategy of commencement of lifelong ART immediately following HIV diagnosis. With ART and even worse without treatment, HIV-1 exposure is associated with adverse birth outcomes and HIV-infected pregnant women have higher rates of stillbirths, preterm births and deliveries of low birth weight infants compared to their HIV-uninfected counterparts [19–25].

ART has greatly improved the outcome of infants with HIV-1 exposure. Without treatment, 31% of HIV-infected mothers transmitted the virus to their infants by 6 months of age and most infants progressed to acquired immunodeficiency syndrome (AIDS) rapidly, with only 50% surviving beyond the age of two [26]. ART has reduced mother to child transmission (MTCT) of HIV-1 to less than 1% in developed countries, and greatly improved infant survival [27]. In Zimbabwe, the Option B+ strategy decreased HIV-MTCT rate to 3.6% in 2014 [28].

In spite of these benefits, concerns have been raised on the growing numbers of HIV-1 exposed uninfected (HEU) infants not achieving comparable health outcomes relative to their HIV-1 unexposed uninfected (HUU) counterparts. HEU infants were more likely to experience growth failure [29–32] and had 1.5-fold higher rates of hospitalisations and 2–3.9-fold higher mortality rates, mostly due to infections [33–37]. Neurodevelopmental outcomes were also worse in HEU children [38], possibly due to exposures to other maternal infections during pregnancy [39, 40] including co-infection with cytomegalovirus (CMV) [41–44].

Antiretroviral drugs can accumulate in the amniotic fluid [45], possibly explaining some adverse outcomes on foetal [46–48] and infant development [49–52]. Dolutegravir is slowly being introduced into clinical practice in SSA. However, it has been associated with a high risk of preterm delivery (31.6%) and birth defects [53, 54].

HEU infants show aberrations of the immune system at birth [55–57] with systemic immune activation [56, 58] which could be linked to adverse health outcomes [59, 60]. HEU infants also have lower antibody titres following vaccination [61, 62] with abnormal B- and T-lymphocyte phenotypic profiles [63, 64]. It is therefore unclear, whether HEU infants are able to mount protective lifelong responses to all vaccines promoted via the national Expanded Program of Immunization (EPI, Table 1).

**Table 1:** The extended program of vaccination (EPI)

Age	Vaccine
At Birth	BCG, (hepatitis b to be introduced soon)
6 weeks	Pentavalent 1, OPV1, pneumococcal 1, rotavirus 1,
10 weeks	Pentavalent 2, OPV2, pneumococcal 2, rotavirus 2,
14 weeks	Pentavalent 3, OPV3, IPV, pneumococcal 3,
9 months	Measles and rubella,
18 months	DTP, OPV booster, measles and rubella.

EPI was established by the WHO in 1974 to develop and expand immunization throughout the world. The Zimbabwe vaccination schedule is indicated. In addition, Vitamin A supplementation is given from 6 months of age and thereafter every 6 months until 5 years. Pentavalent: Diphtheria, Pertussis, Tetanus, Hepatitis B and *Haemophilus influenzae* type B. *IPV* inactivated poliovirus vaccine, *OPV* Oral polio vaccine, *DTP* Diphtheria, *BCG* Bacillus Calmette–Guérin

Taken together, the health of the large and ever growing population of HEU infants is a great public health concern in SSA. There is a complex interplay of maternal-infant HIV and coinfections, ART exposures, immune dysregulation, NCDs and low socio-demographic status associated with food insecurity and poor sanitation. While individual risk factors have been well characterized, there is a need to analyse these parameters in a large comprehensive prospective analysis combining deep clinical, socio-demographic and environmental assessment with extensive bio-sampling, improving the detection of adverse health outcomes. In Zimbabwe, HEU infants have been well-studied during the pre-ART and monotherapy eras [37, 65]. However, contemporary evidence is lacking. We hereby introduce the University of Zimbabwe College of Health Sciences (UZ-CHS) Birth Cohort Study, aiming to address these challenges.

## Methods

### Aim of the study

This study aims to identify predictors for short- and long-term adverse infant outcomes in a resource limited setting. We will examine the impact of maternal HIV infection and comorbidities, cumulative ART exposure *in utero* and throughout the breastfeeding period, including the effects of maternal immune suppression, immune activation and environmental variables.

Our study is focusing on mortality and morbidity of HEU infants, testing established risk factors from previous cohorts done during the pre-ART and monotherapy eras. Data and biomaterials will be collected prospectively from mothers and infants from enrolment at ≥20 weeks of gestation to 2 years after birth to enable capturing of infant development.

### Objectives and outcomes

Primary outcomes:

- The primary outcome of this study is the frequency of stillbirth as well as perinatal and postnatal mortality until two years of age of HEU infants versus HUU infants in a multivariable analysis controlled for confounders.
- Maternal mortality from enrolment until two years after delivery in HIV-infected versus HIV-uninfected participants in a multivariable analysis controlled for confounders.

Secondary outcomes of our study include a range of maternal, birth and infant outcomes summarized in Table 2.

**Table 2:** Secondary outcomes of UZ-CHS Birth cohort study

Secondary outcomes	
1. Comparison of morbidity of HEU vs. HUU infants, defined as impaired growth, immune- and neuro-development and/ or frequent of clinically relevant infections.	
2. To determine any association of ART exposure *in utero* and during breast feeding with pregnancy outcome, infant (HUU, HEU, HEI) growth and immune- and neuro-development.	
3. To determine vertical transmission rates for HIV at birth and within the first 2 years of life and assess risk factors for transmission.	
4. To determine the association between maternal baseline and delivery ART levels in different compartments and infant HIV infection, mortality and morbidity.	
5. To determine HIV drug resistance profiles in mother and infants unresponsive to ART.	
6. To assess abnormalities including lipid profiles, bone, haematological and hepatic toxicities associated with exposure to ART among mothers and infants.	
7. To determine antenatal co-infections including HBV, HCV, CMV, syphilis, intestinal helminths and parasites as single infection or combined infections in HIV-infected and HIV -uninfected - women and determine the impact on pregnancy outcome, infant mortality and morbidity.	
8. To determine the prevalence of infant infectious comorbidities; HBV, HCV, CMV, syphilis, including time points of infections and impact on mortality and morbidity.	
9. To determine the prevalence of maternal NCDs (malnutrition/ obesity, anaemia, diabetes, hypertensive disorders) and determine their impact on pregnancy outcomes, infant mortality and morbidity.	
10. To determine infant non-infectious comorbidities (e.g. anaemia, atopic dermatitis, congenital infections and other conditions) and their impact on mortality and morbidity.	
11. To determine maternal household non-biological factors (socioeconomic state, hygiene) and determine their impact on pregnancy outcome, infant mortality and morbidity.	
12. To determine antenatal reference ranges for MUAC, haemoglobin from FBC analyses, and biochemistry (kidney function, liver function tests, bone, lipid profiles) in pregnant women with a favourable outcome of pregnancy.	
13. To assess delayed neurological development using the Denver II tool in HUU, HEU and HEI infants from 6 weeks to 2 years of age.	
14. To determine humoural immune responses to EPI vaccines (e.g. antibody levels against measles, tetanus, diphtheria, pertussis, polio and rotavirus) in HUU, HEU and HEI infants.	
15. To determine maternal intestinal parasites load using 18S sequencing in pregnancy and relate it to infant immune development and atopy (anti-inflammatory (IL-4, − 5, − 13) and regulatory cytokines (IL-10, TGF-β) at delivery, 6 weeks 48 and 96 weeks of age.	
16. To determine immune activation and systemic inflammatory biomarkers in HUU, HEU and HEI infants at delivery, 6, 48 and 96 weeks of age.	
17. To determine biomarkers for endothelial dysfunction including microbial translocation in HUU, HEU and HEI infants at delivery, 6, 48 and 96 weeks of age.	
18. To determine host genetic markers for infectious disease susceptibility including HLA and KIR gene variants and their association with infection rates of HIV and co-infections in mothers and their infants.	
19. To characterize pathogen genetic diversity including HIV, HBV, HCV, CMV subtypes, prevalent in our study population at time of infection/ earliest available samples and/or at 2 years.	

*ART* antiretroviral therapy, *CMV* cytomegalovirus, *FBC* full blood count, *HBV* hepatitis B virus, *HCV* hepatitis C virus, *HLA* human leukocyte antigen, *HIV* human immune deficiency virus, *HEI* HIV exposed and infected, *HEU* HIV exposed but uninfected, *HUU*: HIV unexposed and uninfected, *IL* interleukin, *KIR* killer immunoglobulin like receptor, *MUAC* mid upper arm circumference, *NCD* non-communicable diseases, *TGF* transforming growth factor

### Study setting

The University of Zimbabwe College of Health Sciences (UZ-CHS) Birth Cohort study is a prospective observational cohort study comparing infants born to HIV-infected and HIV-uninfected women in a resource limited setting of Western high-density suburbs of Harare attending Municipal primary health polyclinics. One thousand two hundred (1200) pregnant women (600 HIV-infected mothers and 600 HIV-uninfected controls) at ≥20 weeks of gestation will be enrolled from February 2016 to June 2019. Participants will be followed as mother-baby pairs (MIPs) at birth, within 10 days of life and at 6, 10, 14, 24, 48, 72 and 96 weeks of age.

Questionnaires will be administered and bio-samples for laboratory tests will also be acquired at each visit. Biomaterials will be acquired for specific tests including tests for infections, clinical biochemistry, full blood counts and immune status, dysbiosis (Tables 3). In addition, at each study visit maternal-infant clinical examinations will be done and used to assess health status including the impact of household and environmental factors. The study will be a non-interventional cohort study design, but participants will be offered health care and health education.

**Table 3:** Summary of the tests done in mothers and infants at different time points

Assessments	Method/threshold value(s)	Equipment/ kits/comment
**HIV-1/2 diagnosis**: All HIV uninfected women at enrolment and every 6 months. All HIV exposed infants from 18 months of age	Qualitative rapid immunochromatographic, whole blood serial testing algorithm according to the national guidelines for HIV testing and counselling [66].	- Initial screening; SD Bioline HIV-1/2 3.0 (Standard Diagnostics Inc., Kyonggi-do, South Korea. -Confirmation; *Alere Determine*™ *HIV-*1/2 (Abbott -Diagnostics, Lake Bluff, USA). -Tie breaker; Western blotting, The Orasure kit, Epitope Inc., USA
**Early infant HIV diagnosis**: All HIV exposed infants from delivery, at every visit until weaning or diagnosis of HIV infection up to 18 months of age [67].	HIV-1 RNA Qualitative Test on dried blood spots (DBS), according to the manufacturer’s instructions.	COBAS1 AmpliPrep/ COBAS1 TaqMan1 HIV-1 RNA Qualitative Test (Roche Molecular System Inc., Branchburg, NJ, USA.
**HIV-1 RNA quantification**: In plasma, of all HIV infected women at enrolment and exit In BM colostrum, and every 6 months All HIV infected infants’ earliest positive DBS sample and at exit	According to the manufacturer’s recommendations. The lower detection limit of the assay; 20 copies/mL	Automated TaqMan Roche Amplicor 1.5 Monitor Test (Cobas AmpliPrep/Cobas TaqMan, Roche Diagnostics, Branchburg NJ, USA.
**Immune status assessment**: All HIV infected women at enrolment and exit All HIV infected infants earliest positive whole blood sample and at exit	CD4^ +^ T-lymphocyte (absolute) count EDTA whole blood sample CD4^+^ T-lymphocyte (percentage).	Partec Cyflow counter (Munster, Germany) be performed within 6 h of whole blood collection.
**Maternal ART levels:** Efavirenz, Lamivudine, Tenofovir will be tested enrolment, and every 12 months in all mothers on ART	Different compartments, plasma, breast milk, urine and amniotic-fluid as previously described [68].	Agilent Technologies, Palo Alto High performance liquid chromatography, Agilent 6430 Triple Quadrupole LC-MS/MS system, CA, USA.
**Syphilis serology**: All mothers at enrolment and exit All exposed infants from delivery and at every visit **HBV/HCV serology:** All mothers at enrolment and exit All exposed infants from delivery and at every visit (DBS) **HBV DNA Assays**: Host genetics of HBV infection susceptibility **CMV serology**: All mothers at enrolment and exit **CMV DNA test**: All mothers at enrolment and exit All exposed infants from delivery and at every visit (DBS) Host genetics of CMV infection/ transmission	Detection of anti-Treponema pallidum antibodies and a non-Treponemal slide agglutination test for the semi-quantitative detection of plasma reagins in serum. HBsAg detection, Profiling; HBsAg, HBsAb, HBeAg, HBeAb & HBcAb Plasma, breast milk HBV DNA for occult hepatitis determination HBV plasma DNA levels HLA, KIR typing (whole blood) CMV IgG, IgM levels, avidity assays. CMV DNA in plasma, amniotic fluid breast milk and urine CMV DNA in DBS HLA, KIR typing	Qualitative rapid immunochromatographic and, SD Bioline 3.0, SD Biosensor, Korea Fortress diagnostics Ltd., UK Screening; SD Bioline, Korea Confirmation; Quality kit, USA Tie breaker: Acon, USA HBV profile All positives: 5 profile (CTK, USA) HBV TaqMan PCR Kit, Norgen Biotek, Norway COBAS1 AmpliPrep/ COBAS1 TaqMan1 HBV DNA Quantitative Test (Roche Molecular System Inc., Branchburg, NJ, USA As previously described (112). PerkinElmer kits, Euroimmun, South Africa RealStar CMV PCR kit v1.0 (Altona Diagnostics, Hamburg, Germany), following isolation of viral DNA using the QIAamp MinElute Virus Spin Kit (Qiagen, Hilden, Germany). As previously described [69].
**Clinical biochemistry**: All mothers serum at enrolment and exit All infants serum within, 6 and 24 months of age	Renal; creatinine, urea, uric acid, Urinary protein and albumin eGFR [70]. Liver enzymes Including AST, GGT, bilirubin, LDH, ALP, Cardiac markers; troponin CKMB. Lipids; total and LDL/HDL cholesterol. Bone profile; calcium, magnesium, phosphate.	Beckman Coulter AU680 chemistry analyser (Krefeld, Germany) Urinalysis strips, Cypress Diagnostics. HELLP syndrome for acute kidney injury assessment LDH: AST ratio > 22 distinguishing TTP from HELLP syndrome. Keiser SD [71]. All results to determine antenatal reference ranges associated favourable outcome of pregnancy
**Full blood counts**: All mothers at enrolment and exit All infants within 10 days of life, 6 and 24 months of age	Maternal haemoglobin concentration < 11.0 g/dl and, and varying with age for infants, < 13.5 g/dl) within the first month of life and < 10.7 g/dl from 9 weeks of age [72].	Mindray© Haematology 3-part differential, 16 parameters BC3600 Analyser (Shenzhen, China) Determine antenatal reference ranges associated favourable outcome of pregnancy
**Maternal blood random glucose and HbA1c:** Once in pregnancy at enrolment	Cut off values for random glucose of 110 mg/dl (6.1 mmol/L) within 2 h of a meal or 101 mg/dl (5.6 mmol/L) more than 2 h postprandial. A cut off for HbA1c of 6.5% will identify undiagnosed diabetes mellitus whilst an HbA1c ≥5.6% will mark individuals with an increased risk for future diabetes [73].	Mindray BS200 Chemistry Analyser (Mindray, Shenzen, China) Determine antenatal reference ranges associated favourable outcome of pregnancy
**Intestinal pathogens:** All mothers at enrolment and exit All Infants at exit	Maternal and infant stool samples will be screened for, *H pylori,* intestinal protozoa and helminths trophozoites and ova.	Direct wet mount microscopy and the formal-ether concentration technique [124].
Humoural immune responses to EPI vaccines Infants At 6 weeks, 12 and 24 months of age	Antibody titres EPI will be measured [74, 75].	The Bio-Plex® **200** system Luminex analyser, Bio-Rad, CA, USA
**Plasma inflammation and cell stress biomarkers**: In a subgroup of MIPs with different durations of exposures to ART from pregnancy to 12 months of age	At least 25 inflammatory chemokines and cytokines measurements.	The Bio-Plex® **200** system Luminex analyser, Bio-Rad, CA USA
**Plasma metabolomics:** > 400 analytes, in a subgroup of MIPs with different durations of exposures to ART from pregnancy to 12 months of age to assess altered amino acid and lipid metabolism	3 platforms -Biogenic amines (Amino acids, catecholamines & polyamines), Signalling lipids (Sphingosine-1-phosphate etc., Positive lipids (Lysophospholipids, phospholipids, cholesterol esters, di/triglycerides & sphingomyelins) as described [76].	UPLC; TQMS; QToF –HPLC; UHPLC –; MS/MS – Triple Quadrupole Mass Spectrometer.
Infants atopic sensitization Infant blood samples from 6, 24 and 96 weeks.	Total and specific serum IgE against food/aero-allergens will be assessed in selected Infants with specific serum IgE levels > 0.3 IU/mL will be considered sensitised [77, 78].	Immuno-assay analyser (Thermofisher, Freiburg, Germany).
Breast milk characterisation Maternal milk from delivery up to weaning time	Micro−/macronutrients, vitamins, oligosaccharides, immunological biomarkers, essential fatty PUFAs such aslinoleic acid and α-linolenic acid: precursors of LCPUFA e.g. arachidonic acid and docosohexaanoic acid, from birth until weaning [79].	Gas chromatography, GC-CP3800, Varian, USA) equipped with a flame ionization detector and a fused silica capillary column 50 m × 0.25 mm (I.D. CP-SIL 88 for FAME), Pennsylvania (PA) 16,823–0048, USA).
**Lab based Sub studies assessments**		
Plasma inflammatory biomarkers In a subgroup of MIPs from pregnancy, breastfeeding period and after weaning	−7 panels, including cytokines, chemokines, vascular injury, angiogenesis, Th17.	V-PLEX Human Biomarker 54-Plex Kit, ECL Technology, Maryland, USA.
Multiplexed 16S sequencing In a subgroup of MIPs from pregnancy, breastfeeding period and after weaning	16S rRNA gene segment spanning V5 and V6 regions in stool, plasma and breast milk as previously described [80].	Illumina MiSeq/Ion Torrent PGM sequencer.
Breast milk (whole) immune-metabolomics In MIPs with adverse outcomes, from colostrum, and at least 3 later points.	Non-targeted injection analysis.	Mass spectrometry (TQF-MS), Agilent 6550, Agilent Technologies Inc., Santa Clara, CA.
Maternal mycotoxins biomarkers In pregnancy, at delivery and exit	Level of mycotoxin biomarkers including aflatoxins B1 and 2 in urine, amniotic-fluid and milk	Measurement will be performed on Agilent 6430 Triple Quadrupole LC-MS/MS system, Palo Alto, CA, USA
Natural killer (NK) cells characterisations In a subgroup of HEU (with short, medium and long term ART exposures) versus HUU infants two time points between 12 and 24 months of age.	NK cells surface marker staining: CD2, CD3, CD16, CD56, CD69, KIR and mitochondria functional assays following PBMC isolation, including nutrient transporter expression on NK cells: Glut1, CD98, and CD71. Mitochondrial function of NK cells will be done using mitotracker green and mitotracker deep red assays.	The BD LSR Fortessa™ cell analyser, multicolour (16 colour) flow cytometer assays, BD Sciences
Maternal latent TB testing: In a subgroup of HIV infected and uninfected in pregnant women and 6 weeks postpartum	CD4/CD8 IGRAs against TB antigens (ESAT-6/CFP-10/TB-7.7 against TST/ Mantoux at 0.1 ml dose.	Qiagen QuantiFERON gold plus kit, USA, Tuberculin purified protein derivative (PPD) Mantoux, Tubersol^R^, Sanofi Pasteur, in tween solution, 5 US units per test
Salivary cortisol levels In a subgroup of MIP at 6 months follow up visit	Test done according to the manufacturer’s recommendations. Sensitivity is 0.007μg/dl.	Salimetrics, LLC competitive enzyme immunoassay kit, Carlsbad, CA USA.
***Helicobacter pylori*** **testing** In a subgroup of MIP from pregnancy to exit	Quantitative ELISA; *H. pylori* IgG IgA, IgM antibodies and detection of *H pylo*ri antigens from birth to 24 months according to the manufacturer’s recommendations *H pylori* Ag Sensitivity:0.165 ng/ml.	Sandwich enzyme Immunoassay, Colorimetric test ELISA**,** Monobind Lake Forest, CA USA.

*ALP* alkaline phosphatase, *AST* aspartate aminotransferase, *BM* breast milk, *CD* cluster of differentiation, *CK-MB* creatine kinase-myocardial band, *CMV* cytomegalovirus, *EPI* expanded program for immunization, *GC* gas chromatography, *GGT* gamma-glutamyl transferase; *Glut1* glucose transporter-1, *HbA1c* glycosylated haemoglobulin, *HBcAb* hepatitis B core antibody, *HBeAb* hepatitis B e-antibody, *HBeAg* hepatitis B e-antigen, *HBsAb* hepatitis B surface antibody, *HBsAg* hepatitis B surface antigen, *HBV* hepatitis B virus, *HCV* hepatitis C virus, *HDL* high density lipoprotein, *HEI* HIV exposed infected, *HELLP* haemolysis, elevated liver enzymes and low platelet count, *HEU* HIV exposed but uninfected, *HIB Haemophilus influenza* type b, *HIV* human immunodeficiency virus, *HLA* human leukocyte antigen, *HPLC* high-pressure liquid-chromatography, *HUU* HIV unexposed and uninfected, *IGRAs* interferon gamma release assays, *KIR* killer cell immunoglobulin-like receptor, *LCPUFA* long chain poly unsaturated fatty acids, *LDH* lactate dehydrogenase, *LDL* low density lipoprotein, *MIPs* mother-infant pairs, *MS* mass spectrometer, *PUFA* poly unsaturated fatty acids, *QToF* quad time of flight, *TB* tuberculosis, *TQMS* triple quadrupole mass spectrometer, *TST* tuberculin skin test, *TTP* thrombotic thrombocytopenic purpura, *UHPLC* ultra-high-pressure liquid-chromatography, *UPLC* ultra-pressure liquid-chromatography

### Study sites, local municipal engagement and routine clinical care

The Municipality of Harare has established 12 polyclinics each comprising units for primary health care, maternity, post-natal care and a family health service. Medical services include HIV counselling and rapid testing including initiation and administration of ART. Monitoring and follow-up for other chronic conditions including diabetes mellitus and arterial hypertension are also offered. A physician comes twice a week per site to attend to complicated cases. Maternity services comprise antenatal care, postnatal care, treatment of hypertensive disorders, HIV and syphilis screening and treatment.

For each pregnant woman, routine lab results and management prior to enrolment into the study are well documented in antenatal booklets during routine clinical care. The study team will have access to these clinical data during and after the enrolment visit. The standard of care for all Zimbabwean HIV-infected women to prevent HIV-MTCT consists of (non)–nucleoside reverse transcriptase inhibitors; TENOLAM-E (Tenofovir, Lamivudine and Efavirenz, and to breastfeed as long as they choose to. Exclusive breast-feeding is encouraged during the first six months of life, with breastfeeding along with appropriate complementary foods up to 2 years of age or even older. According to Zimbabwean national guidelines, all HIV-infected women should be on Option B+, but in practice women often do not receive a timely HIV diagnosis.

Independent from our study, all infants including HIV-1 exposed and infected (HEI) infants, are monitored for growth, and also receive vaccination as shown in Table 1 as per the Zimbabwean EPI program. Clinic visits for any ailments are managed at a local primary health centre with complicated cases being appropriately referred to the nearest tertiary hospital. All HIV exposed infants are tested for HIV by reverse transcriptase polymerase chain reaction (RT-PCR) (Table 3, point ii.) at birth, 6 weeks of age and after cessation of breast feeding. Upon detection of HIV infection, infants are immediately commenced on ART, usually comprising the protease inhibitors Lopinavir/ritonavir and the nucleoside reverse transcriptase inhibitors; Zidovudine and Lamivudine. In addition, all HIV exposed infants are commenced on Cotrimoxazole prophylaxis until they stop breast feeding or they test HIV RT-PCR positive, whichever occurs first.

It costs a pregnant woman United States (US) twenty-five dollars ($25) to access antenatal services until six weeks after delivery whilst health care services are free of charge for infants until 5 years of age. They are encouraged to book early during pregnancy (at week 4–12 gestational age) and to test for HIV together with their spouses/partners. Currently, due to social-economic challenges, most Zimbabwean pregnant women go for antenatal registration at the polyclinics when their pregnancies are at advanced stages, a situation that puts their unborn babies at risk for HIV transmission, perpetuating the vicious cycle of poverty and HIV infections.

Following a pre-study site feasibility assessment in September 2015, out of the 12 polyclinics, Kuwadzana, Dzivaresekwa (Rujeko), Glenview and Budiriro in South West of Harare city centre were selected based on the higher volumes of maternal and child health services, frequency of HIV positivity and lack of competing research activities targeting the same population. The catchment areas of the selected four polyclinics have relatively stable communities that do not frequently change their rented accommodation. For this study, Municipal midwives at the selected facilities will assist with sample/data collection for deliveries that will occur late at night.

### Background characteristics of study participants

All participants reside in South-Western high-density areas of Harare. Residents of this community are of relatively poor socio-economic status with high unemployment rates. Most households generally cannot afford 3 decent meals per day. Most families (72%) live on less than 1 dollar per day [81] and supplement their diet through small scale vegetable gardening around their houses. Families live in houses of similar structure and size (200 m^2^). On average 2–6 families live in these relatively small houses and in some extreme situations up to 5 family members share a single room. Water supply is erratic, necessitating the use of communal boreholes at 200–1500 metres distance. This manually fetched water is used for drinking, cooking, bathing and flushing of toilets. Sewage bursts are common and may occur close to these boreholes. Contaminated boreholes in two of our four study sites, Budiriro and Glenview, have been epicentres of the 2018 cholera epidemics [7]. Residents of these high-density residential areas share a disproportionate burden of infectious diseases relative to those living in low-density areas in the same city.

### Study participants

UZ-CHS birth cohort study enrolled consenting pregnant women registering for antenatal care at any of the 4 selected City of Harare polyclinics. Upon delivery, babies will be automatically recruited into the study and followed up as MIPs for two years. In cases of multiple births (twins or triplets) all infants of the recruited mother will be followed. Follow up is feasible and relatively easy in Zimbabwe where about 90% of pregnant women own or have access to a mobile phone [82].

### Inclusion and exclusion criteria

Pregnant woman of Bantu origin should be ≥15 years of age, at least 20 weeks of gestation at enrolment and planning to deliver at any of the 4 study sites. Due to the current economic challenges, nearly all pregnant women in high-density areas in Harare present to Municipal health clinics at or beyond 20 weeks of pregnancy. Therefore, no upper limit for gestational age for inclusion applies, to ensure a representative study population. All pregnant women with a documented positive HIV status will be encouraged to enrol in the study. For every HIV-infected pregnant woman recruited, the 10th HIV-uninfected woman presenting to the same primary health clinic for the initial antenatal registration will be recruited, accounting for the 12% HIV prevalence within this population. The pregnancy will be dated by the standard way of using menstrual history together with ultrasound scans whenever available as documented in the antenatal booklet which is routinely issued to all pregnant women. Mothers should be willing to be followed together with their babies from delivery, and willing to provide the required data and biological specimens in follow-up visits for two years. Exclusion criteria will be the presence of severe maternal mental health disorders, rendering the women incapacitated to provide informed consent or comply with study procedures as assessed by the study Clinicians.

### Sample size and statistical power

The composite primary endpoint of our study is the comparison of stillbirths and mortality in HEU versus HUU children until two years of age. In Zimbabwe, infant mortality rates of 50 per 1000 live births in the first year and 5 deaths per 1000 live infants in the second year of life are expected. In the pre-ART era, a 3.2–3.9-fold higher mortality in the first year of life and a 2-fold higher mortality in the second year of life was observed [37, 83]. Mortality of HEU in the Option B+ era with more effective HIV treatment might now be improved and in a conservative estimation, we assumed a 2-fold increase in mortality in HEU infants within the first two years of life.

We therefore expect 5.5% mortality in HUU infants and 11% in HEU infants. According to our power analysis using G*Power3 [84], a sample size of 1198 pregnant women will be required to detect such a difference with a power of 90% and a significance level of 0.05 in a two-sided analysis. We therefore, will opt for a sample size of 1200 pregnant women, 600 with and 600 without HIV infection.

### Selection of participants

Potential participants will be identified during routine antenatal care visits at any of the four selected City of Harare Polyclinics. The potential participants will be briefed on the study and those who would have verbally agreed to participate in the study will be given the participant information sheet in the language of their choice, either in English or vernacular Shona to ensure full comprehension of the study aims and expected activities using the language that the participant understands best. Participation is voluntary and women will be free to withdraw at any time during the course of the study. Literacy is nearly universal in Zimbabwe [85] and all potential participants should be able to read and comprehend the informed consent form. However, in cases of illiterate study participants, consent will be documented by clearly impressing the right thumbprint on the signature page of the consent form. The actual process of right thumb printing will be witnessed by the study participant’s literate relative of her choice who will print his or her name on the informed consent form and will also sign as a witness in the presence of the study team member.

No payment to research participants is required by Zimbabwean law and hence no formal payments to participants will be made for this study. However, research participants will be reimbursed US $5 to cover travel expenses at every study visit and offered refreshments during the waiting time that will range from 30 to 60 min. The study team will offer health education and counselling on pregnancy, delivery and childcare on a continuous basis on each study visit.

### Mothers’ questionnaires procedures and measurements

A midwife will administer a structured questionnaire to all pregnant women at enrolment aiming at a comprehensive clinical, behavioural and environmental characterization. Specific questions will address sexual behaviour and reproductive health issues including sexually transmitted diseases and contraception use. General health, health seeking behaviour, maternal stress and drug/herbal use will be assessed. To address hygiene, specific questions on sanitation (type and number of toilets, sewage system), drinking water, type of energy used (indoor pollution), type of floor(s) in the house, number of rooms used for sleeping by the family and the number of individuals sharing a single room will be asked. Economic information comprising employment status, family monthly income, food security, diet, monthly money set aside for food, healthcare, cooking fuel and savings will be assessed. Ownership of agricultural land or household assets including radios, televisions, mobile or fixed telephones, refrigerators, bicycles, motorcycles or scooters, and cars will provide additional information regarding social economic status. Further questions will address maternal life style such as alcohol use, smoking, sleeping habits, physical activities, domestic violence and support from spouse/partner including knowledge, beliefs and practices in relation to patterns of seeking health care services, see Tables 4 and 5.

**Table 4:** Maternal measurements and data collection time points

Variables	Research visit (in weeks)										
	Pregnancy	Delivery	1	6	10	14	24	36	48	72	96
**A. Maternal life style**											
1. Current alcohol use	✓			✓			✓				✓
2. Smoking											
3. Living under same roof with spouse/partner	✓			✓			✓				✓
4. Current marital/ domestic violence	✓			✓			✓				✓
5. Physical activity	✓						✓				✓
**B. Household factors**											
1. Household numbers & composition	✓			✓			✓				✓
2. Number of rooms available	✓			✓			✓				✓
3. Main source of drinking water	✓			✓			✓				✓
4. Main source of energy for cooking (household pollution)	✓			✓			✓				✓
5. Main place for cooking	✓			✓			✓				✓
6. Sleeping arrangements	✓			✓			✓				✓
Water and sanitation	✓			✓			✓				✓
7. Presents of family diarrhoeal diseases	✓			✓			✓				✓
**C. Nutritional factors**											
1. Maternal number of meal a day	✓			✓			✓				✓
2. Household meals eaten per day	✓			✓			✓				✓
3. Food frequency questionnaire	✓			✓			✓				✓
4. Food security tool	✓			✓			✓				✓
5. Perceived food allergens/ adverse reactions	✓			✓			✓				✓
6. 24 h food recall	✓						✓				✓
7. Craved foods	✓										
8. Any disorder related to food mastication and/ or retention	✓			✓			✓				✓
9. Bristol stool chart	✓										✓
**D. General health**											
1. Use of condom and sexual behaviour	✓			✓			✓				✓
2. Past and current methods of contraception used	✓	✓		✓			✓				✓
3. 7Health seeking behaviour	✓			✓			✓				✓
4. Tb symptoms checklist for active Tb	✓	✓		✓			✓				✓
5. History of Tb disease and treatment	✓						✓				✓
6. Current and past other opportunistic infections	✓			✓			✓				✓
7. Past and current symptoms of sexually transmitted infections	✓	✓		✓			✓				✓
8. Past or current hypertension	✓	✓		✓			✓				✓
9. Past or current stress	✓	✓		✓			✓				✓
10. Baby blues assessment tool		✓									
11. Depression	✓	✓					✓				✓
12. Hospitalisation in the past 3 months	✓			✓			✓				✓
13. Sick clinic visits during index pregnancy	✓										
14. Vaccinations during index pregnancy	✓										
15. Timing of antenatal booking, number of subsequent visits	✓										
16. Knowledge of pregnancy disorders that warrant immediate hospitalization	✓										
17. Concurrent medication and supplements	✓	✓		✓			✓				✓
18. Concurrent herbal and traditional medication	✓	✓		✓			✓				✓
19. Use of sexual enhancing herbs/ substances	✓						✓				✓
20. History of routine dental or pap smear checks	✓						✓				✓
21. Anthropometric measurements (BMI, MUAC))	✓	✓	✓	✓	✓	✓	✓				✓
22. Systolic and diastolic BP and pulse measurements	✓	✓	✓	✓	✓	✓	✓				✓
23. General physical examination (e.g. oedema)	✓	✓	✓	✓	✓	✓	✓				✓
24. Last date of menstruation											
**E. HIV-infected only: ART use, duration, disclosure & adherence**											
1. ART use regimen, duration, switching of regimens	✓	✓	✓	✓	✓	✓	✓	✓	✓	✓	✓
2. Presence of adequate supplies	✓	✓	✓	✓	✓	✓	✓	✓	✓	✓	✓
3. ART side effects	✓	✓	✓	✓	✓	✓	✓	✓	✓	✓	✓
4. Non-adherence issues	✓	✓	✓	✓	✓	✓	✓	✓	✓	✓	✓
5. Disclosure of HIV status	✓	✓	✓	✓	✓	✓	✓	✓	✓	✓	✓
6. Stigma experience	✓	✓	✓	✓	✓	✓	✓	✓	✓	✓	✓
7. WHO clinical staging for the HIV-infected	✓	✓	✓	✓	✓	✓	✓	✓	✓	✓	✓
**F. Clinical data collected at delivery**											
1. Mode of delivery		✓									
2. Duration of labour		✓									
3. Live and stillbirths											
4. Term/ preterm delivery											
5. Pregnancy outcome		✓									
6. Length of stay in hospital/ clinic after delivery		✓									
7. Obstetric related complications											
8. Time of previous ART intake for HIV-infected mothers		✓									
**G. Biological specimen collection in the 3rd trimester**											
**1. Stool**	✓										
i) Intestinal helminths testing											
ii) Microbiome profiles (sub-studies)											
**2. Urine** i) Urinalysis and storage microbiome	✓	✓	✓	✓	✓	✓	✓	✓	✓	✓	✓
**3. Blood**											
i) Serology, molecular genetics, HIV, CMV, HBV, syphilis	✓										✓
ii) TB QuantiFERON gold (sub-study)	✓			✓							
iii) Full blood counts	✓										✓
iv) Infant vaccine titres	✓										✓
v) Biochemistry	✓										
a. Dyslipidaemia	✓										✓
b. Kidney function tests	✓										✓
c. Liver function tests	✓										✓
i) Vitamin D	✓										✓
ii) HbA1c, random glucose	✓										
iii) Cytokine profiles	✓	✓	✓	✓	✓	✓	✓	✓	✓	✓	✓
iv) CD4 counts	✓										✓
v) HIV RNA load	✓				✓						✓
vi) CMV DNA load	✓										✓
vii) HBV DNA load											
viii) Host genetic susceptibility											✓
ix) Immunometabolomics	✓	✓	✓	✓	✓	✓	✓	✓	✓	✓	✓
**H. Delivery bio-specimens**											
1. Placental pathology in latent Tb (sub-study), microbiome		✓									
2. Amniotic-fluid co-infections, alcohol levels, microbiome		✓									
3. Breast milk (lipid profiles and microbiome, CMV viral load), microbiome		✓	✓	✓	✓	✓	✓	✓	✓	✓	✓
**I. Spouse/ intimate partner factors (as reported by mothers)**											
1. Age	✓										
2. Occupation	✓										✓
3. Previous marital status	✓										
4. HIV status, ART usage	✓										✓
5. Participation in pregnancy, ante	✓										✓
6. Participation in child care	✓			✓							✓

*ART* antiretroviral therapy**,** *BMI* body mass index, *CD4* cluster of differentiation-4, *CMV* cytomegalovirus, *EPI* The expanded program of immunisation, *MUAC* mid upper arm circumference, *HbA1c* glycated haemoglobin, *HBV* hepatitis B virus, *HCV* hepatitis C virus, *HLA* human leukocyte antigen, *HIV* human immunodeficiency virus, *KIR* killer cell immunoglobulin-like receptor, *SGA* small for gestational age, *TB* tuberculosis, *WHO* world health organisation

**Table 5:** Infant data and health assessment tool

Exposure and data collection points (delivery=0 time point)	Research visit (weeks)									
	0	1	6	10	14	24	36	48	72	96
**a) Infant in utero exposures**										
1. HIV exposure in utero/ postpartum	✓	✓	✓	✓	✓	✓	✓	✓	✓	✓
2. Antenatal dysbiosis, microbiome	✓	✓	✓	✓	✓	✓	✓	✓	✓	✓
3. Congenital co-infections; HBV, HCV, CMV, syphilis	✓	✓	✓	✓	✓	✓	✓	✓	✓	✓
4. Alcohol exposure (foetal alcohol syndrome)	✓	✓	✓	✓	✓	✓	✓	✓	✓	✓
5. Food quantity and quality, antenatal diet, maternal BMI	✓	✓	✓	✓	✓	✓	✓	✓	✓	✓
6. Allergens, food, animal dander	✓	✓	✓	✓	✓	✓	✓	✓	✓	✓
7. Indoor pollution (paraffin stove cooking in one room/smoker)	✓	✓	✓	✓	✓	✓	✓	✓	✓	✓
8. Household factors water/sanitation (quantity/quality)/	✓					✓				✓
**b) Delivery & Postpartum Data**										
1. Delivery date, gender, SGA, Apgar score, congenital abnormalities	✓									
2. Weight, head circumference and length (MUAC from 6 weeks)	✓	✓	✓	✓	✓	✓	✓	✓	✓	✓
3. Infant morbidity, sick clinic visits, hospitalisations, atopic dermatitis	✓	✓	✓	✓	✓	✓	✓	✓	✓	✓
4. Infant mortality	✓	✓	✓	✓	✓	✓	✓	✓	✓	✓
5. EPI immunisation compliance	✓	✓	✓	✓	✓	✓	✓	✓	✓	✓
6. Any medication being taken	✓	✓	✓	✓	✓	✓	✓	✓	✓	✓
**c) Infant developmental assessment tools**										
1. Denver tool			✓	✓	✓	✓	✓	✓	✓	✓
2. Mullen tool (neurodevelopmental sub-study)						✓	✓	✓	✓	✓
**d) Biological specimen collection**										
**1. Cord blood**	✓									
2. Blood and dried blood spots										
a) Vertical transmission of HIV, CMV, HBV, syphilis	✓	✓	✓	✓	✓	✓	✓	✓	✓	✓
b) EPI vaccine titres			✓		✓			✓		✓
c) Plasma immune activation biomarkers	✓	✓	✓	✓	✓	✓	✓	✓	✓	✓
d) Systemic plasma Inflammation biomarkers and trends	✓	✓	✓	✓	✓	✓	✓	✓	✓	✓
e) Endothelial dysfunction biomarkers	✓	✓	✓	✓	✓	✓	✓	✓	✓	✓
f) Immunometabolomics	✓	✓	✓	✓	✓	✓	✓	✓	✓	✓
g) Biomarkers of intestinal structure and function (permeability, microbial translocation)	✓	✓	✓	✓	✓	✓	✓	✓	✓	✓
h) Food allergens and atopic dermatitis sensitisation trends	✓	✓							✓	✓
i) Full blood counts and clinical biochemistry			✓				✓			✓
j) HIV viral load (in infected individuals)	✓	✓	✓	✓	✓	✓	✓	✓	✓	✓
k) Pathogen genetic diversity and Host infection genetics	✓	✓	✓	✓	✓	✓	✓	✓	✓	✓

*BMI* body mass index, *CMV* cytomegalovirus, *EPI* The expanded program of immunisation, *MUAC* mid upper arm circumference, *HBV* hepatitis B virus, *HCV* hepatitis C virus, *SGA* small for gestational age

In HIV-infected mothers, ART use, regimen and date for commencement, co-infections and issues related to HIV status disclosure and stigma will be recorded. The information will be verified using clinical records in the maternal antenatal booklet.

A significant fraction of HIV-infected women will be unaware of their HIV status and therefore will be ART naïve [86]. In these women, ART will be initiated upon first presentation at the antenatal clinic, coinciding with enrolment into our study. Due to the unfortunate scenario of late antenatal bookings some women will therefore commence ART late or very late in pregnancy during labour. However, other women will be on ART even before conception. This provides the opportunity to study the impact of different durations of ART exposure (as a continuous variable based on gestational age at ART initiation) on birth and infant outcomes. Other than the *in utero* exposure, infants’ exposure to ART will continue throughout the breastfeeding period since mothers are encouraged to breastfeed regardless of their HIV status.

### Physical examination

At enrolment, the study midwife will perform a full physical examination, blood pressure checks, assessment of oedema and anthropometry. World health organisation (WHO) clinical staging will be done for all HIV-infected women. The examination will also include measurement of symphysis fundal height (SFH) for calculation of gestational age in comparison with last menstrual period (LMP) that is subject to considerable margin of error, particularly late in pregnancy. The SFH will be determined by measuring the distance in centimetres (cm) from the pubic symphysis to the highest part of the uterus.

Assessment of nutritional status will be done using standard anthropometric indices including body mass index (BMI). Mid upper arm circumference (MUAC) is an easy, non-invasive and inexpensive technique in anthropometry to detect nutritional status [87]. MUAC is considered an indicator of the body protein reserves, and unlike BMI is independent of gestational age [88]. MUAC will be used as an indicator for malnutrition, including obesity. Mother’s left arm will be bent at the elbow at a 90 degree angle, with the upper arm held parallel to the side of the body. The upper arm circumference will be measured at the mid-point distance between the bony protrusion on the shoulder (acromion) and the point of the elbow (olecranon). Previous South African studies demonstrated that a MUAC of < 23 cm, predicted a pre-pregnancy BMI of < 18.5 kg/m^2^ and a MUAC of > 30.57 cm, predicted a pre-pregnancy BMI of > 30 kg/m^2^ [88]. For more practical reasons, a maternal MUAC cut-offs of ≤25 cm will be used as a marker for malnutrition. Blood pressure (BP), both systolic and diastolic will be measured after allowing the mother to rest for at least 5 min.

### Maternal and infant follow-up visits

Mothers will be reminded of their upcoming follow-up study visits by phone call or through text message. Follow-up will be scheduled at delivery, within 10 days, 6, 10, 14, 24, 36, 48, 72 and 96 weeks postpartum. If a mother fails to turn up for their scheduled follow-up appointments, attempts to contact her or her next of kin by phone and/ or home visits will be made strictly adhering to the documented mother’s preferences. Data and samples will still be collected provided the missed visit can be rearranged within the following seven days. In case of two consecutive missed visits without communication, study discontinuation will be discussed with the mother.

Information on place, date and mode of delivery will be extracted from “Road to Health Child’ cards” issued at birth for monitoring growth, vaccinations records until 5 years of age. Longitudinal infant feeding practices and comprehensive information on health, development and environment will be collected. Extensive questionnaires similar to the one administered at enrolment will be re-administered at 6, 12, 18 and 24 months. Physical examinations of mothers will be performed until week 6 after delivery and at exit. For infants physical examinations will be done at every visit. Sick infants will be seen by the study Paediatrician. Study procedures also include bio-sampling of blood, breast milk and stool at every study visit.

### Adverse birth outcomes and infant mortality

The key outcome of this study is mortality of infants assessed by the composite endpoint of stillbirth (gestational age at birth ≥20 weeks) and infants who die during the first two years of life.

Adverse birth outcomes will include the need for resuscitation at birth due to birth asphyxia or respiratory distress syndrome. These conditions can be accurately diagnosed by a midwife. Birth asphyxia will be defined as any medical condition resulting in decreased or discontinued supply of oxygen to a new-born infant before, during or soon after birth. No formal paediatric assessment will be performed at birth; therefore, accurate diagnosis of other paediatric conditions will not be feasible at this point. Other adverse outcomes at delivery will include preterm birth. WHO criteria will be applied in calculations regarding infants’ anthropometric measurements [89]. Gestational age will be divided into early preterm (20–31 weeks), late preterm (32–36 weeks), term (at least 37 weeks) and post term birth (> 42 weeks of gestation). Additional adverse outcomes are a low Apgar score at 5 min (< 7), small for gestational age (SGA, i.e. weight/ length below the 10^th^ percentile for gestational age), low birth weight (LBW) (< 2500 g, weighed within the first hour of life, before significant postnatal weight loss would have occurred), very LBW (< 1500 g), extremely low birth weight (< 1000 g), foetal macrosomia (birth weight >  4000 g), microcephaly (head circumference < 2 standard deviation (SD) from the mean) and multiple births. A brief physical examination will be performed by the midwife regarding structural birth defects (malformations, e.g. extra digits). All infants with suspicious findings regarding birth defects will be followed up by a team of four board certified study Paediatricians.

### Infant growth

Growth will be expressed as Z scores according to WHO definitions [89]: Weight-for-age (WAZ), height-for-age (HAZ), weight-for-height (WHZ) and head-circumference-for-age (HCAZ). Growth outcomes will be evaluated as continuous variables (attained Z-score and change in Z-score between visits). In addition, the following categorical outcomes will be assessed:

- Moderate wasting, WHZ < –2
- Stunting, HAZ < –2
- Severe stunting, HAZ < –3
- Underweight, WAZ < –2
- Microcephaly, HCAZ<-2.

Infant MUAC will be measured from 6 weeks of age and sensitivity, specificity, positive predictive values, and negative predictive values of the MUAC for stunting, underweight, wasting, anaemia and adverse neurodevelopment in HEI, HEU and HUU infants aged 0- < 6 months, 6- < 12 months and 12–24 months will be determined. WHZ will be used as the gold standard for wasting.

### Assessment of infant morbidity

Morbidity diaries will be issued to mothers at delivery. Medical professionals unrelated to the study (e.g. Municipal primary health nurses) will also document medical reports in the morbidity diary during sick visits and/or hospitalizations. These will include: neonatal hypoglycaemia, neonatal jaundice, infant anaemia, skin rashes, congenital infections, vertical transmission of infections over the 2 years, any hospitalisation (frequency and duration), sick visits (dates and duration), documented local inpatient treatments, stunting, wasting and abnormal neurodevelopment. After episodes of illnesses, follow up notes will be recorded in the diary by the study nurse. The mother will also continuously assess infant well-being and will be encouraged to document any perceived child sickness in the morbidity diary. Contents of morbidity diaries will captured, pictured/photocopied at each study visit.

### Infant development

Neurodevelopmental outcomes will be evaluated from six weeks until two years of age using the Denver II screening tool [90]. The Denver II tool enables assessment of gross and fine motoric abilities, development of language, social skills, attention span, general compliance and fearfulness. In addition, infant developmental milestones (e.g. timing of teething, first sitting, walking or talking) will be recorded. No neurological assessment tool has been formally validated in Zimbabwe; the Denver tool will be used since it can be conveniently applied by the study nurse [90]. Our study Paediatricians’ clinical experiences indicate that the tool is appropriate without adjustment for the Zimbabwean setting. For detailed neurodevelopmental and cognitive assessment, the Mullen tool [91] will be used in a sub-study at 12 and 24 months of age.

### Maternal biosamples

Biological specimens including whole blood, plasma and serum, stool, and breast milk will be collected at all visits; cord blood, amniotic fluid and placenta will be collected at delivery. Sensitive samples (stool, milk, urine) will be placed at 4 degree Celsius (°C) for no longer than 4 h before processing. All samples will be stored in a bio-bank at − 80 °C within a maximum of 6 h after acquisition.

Different lab tests will be done (Table 3). Most tests will apply for all mothers regardless of their HIV status for comparison of disease burden between both groups.

HIV-uninfected mothers will be continuously counselled and retested for HIV infection every 6 months until 2 years after delivery (Table 3 point i.). Sero-converting women will be referred to the national HIV prevention program for immediate commencement of ART and immunological and virological monitoring.

### Infant blood samples

Infant venous blood will be collected whenever possible at 7–10 days and 24 months for full blood counts. Whenever possible, plasma samples will be stored. The volume of blood collected will be within recommended amounts depending on the weight of the infant (i.e. ≤1–5% of total blood volume over 24 h and ≤ 10% over 8 weeks) [92]. Blood will not be collected from severely sick infants and those with suspected severe anaemia according to clinical assessment.

Dried blood spots (DBS) will be collected wherever possible at every visit using whole blood samples or a needle prick puncture of the heel. 3–5 drops of blood will be collected and dried on Whatman filter paper and stored at − 20 °C.

HIV exposed infants will also be tested for HIV within 10 days of life, at weeks 10, 14, 24, 36, 48, 72 and 96 or until cessation of breast feeding, whichever comes first.

### Summary of sub-studies

#### Gut microbiota sub-study (longitudinal analysis)

Chronic intestinal maladaptive and inflammatory alterations in a resource limited setting with inadequate nutrition and hygiene are summarized as environmental enteric dysfunction (EED). EED is characterized by a delayed and aberrant maturation of the intestinal microbiota [93, 94], ultimately resulting in dysfunction and failure of the microbiota metabolic organ [95]. Understanding microbiota maturation will likely be key to the pathogenesis of EED. HIV infection and ART use have also been associated with a disturbed gut microbiota [96] and might further aggravate EED.

For in-depth microbiota analysis, sequential stool samples taken from MIPs will be used to perform 16S rRNA sequencing for assessment of phylum and operational taxonomic unit composition with ≥97% sequence identity, as well as microbial diversity [97]. In selected samples bacterial full genome metagenomics shotgun sequencing to identify the microbiota metabolic potential (i.e. bacterial genes present [96] will be done. Further, mass spectrometry will be performed for analysis of small intestinal content (metabolome) (83;84) including assessment of markers of microbial translocation across the damaged gut wall [98] such as lipopolysaccharide (LPS), a component of gram-negative bacterial cell walls, a biomarker of microbial translocation [99], with intestinal fatty acid binding protein (I-FABP) as a biomarker for enterocyte damage [100]. Continuous passage of immuno-stimulatory LPS from the intestinal lumen into the systemic circulation sustains activation of both innate and adaptive immunity causing systemic immune activation through activation nuclear factor kappa beta (NF-ϰβ) protein complex that controls transcription of DNA in cytokine production [101]. Protective factors include immunoglobulin M (IgM), IgG, and IgA specific for the LPS core antigen and endotoxin core antibodies (EndoCAb) [102]. Microbial translocation has been associated with HIV clinical progression and early onset of non-AIDS comorbidities. How microbiota profile is affected by HIV /ART exposures and cotrimoxazole prophylaxis in settings of poverty and malnutrition remains to be explored with respect to infant growth, development and susceptibility to infectious diseases. Use of probiotics and prebiotics can be potentially used to modify the disturbed /imbalanced bacterial profile in the gastrointestinal tract of HEU infants. This microbiota sub-study will involve sampling of ≥100 women in pregnancy and MIPs within 10 days of life, at weeks 6, 10, 14, 24, 36, 48, 72 and 96. Biosamples include MIPs plasmaand stool samples, including breast milk. See Table 3**xxi, xxii, xxiii and xxiv** for details.

#### Immuno-metabolomics sub-study (longitudinal analysis)

Interference with lipid, glucose amino acids metabolism is another mechanism for ART toxicity. This sub-study aims to investigate plasma inflammatory immune activation markers as risk factors for adverse pregnancy outcomes in HIV-infected women and subsequent impaired infant growth and development. For this study-study we will select a sub group of HIV-infected pregnant women with long-term (> 7 months), medium-term (1–7 months) and no/ short-term (no or < 1 months) ART use and uninfected women as controls. We will test for systemic inflammatory immune activation markers in MIPs plasma samples at enrolment, delivery, 10 days, 6, 14, 24, 48 and 72 weeks. Enzyme linked immuno assay (ELISA)-based multiplex testing by Luminex technology will be used to measure chemokines and cytokine levels including, B cell-attracting chemokine 1, brain-derived neurotrophic factor, interferon gamma inducible protein 10, monokine induced by gamma interferon, monocyte chemo-attractant protein 1, macrophage inflammatory protein-1β, tumour necrosis factor alpha, matrix metalloproteinase-1, transforming growth factor beta, interferon gamma, interleukin (IL)-1β, IL-2, IL-4, IL-6, IL-7, IL-10, IL-12, IL12p70, IL-17A, IL-27, INF-γ, cluster of differentiation (CD) 30 and CD40. See Table 3**xxviii** for details.

To assess the metabolome and mitochondrial dysfunction, mass spectrometry techniques will be used to measure a wide range of metabolites including reactive oxygen species, triglycerides, diacylglycerides, cholesterol, phospholipids, fatty acids, peroxidized lipids and oxylipids. Metabolite levels will be correlated to pregnancy outcome and with infant growth (LBW, SGA, stunting wasting) and infants’ immune responses to vaccines. This metabolomics component will involve convenient sampling of *≥*55 women in pregnancy and respective MIPs within 10 days of life, at weeks 10, 14, 24, 36 and 48. Biosamples include MIPs plasma samples. See Table 3**xxix** for details.

#### Cell-mediated immunity/ glucose metabolism sub-study

HIV infects activated but not resting CD4^+^ T-lymphocytes, and hence the need to understand how host nucleotide, glucose, lipid, amino acid metabolism including the respective cell oxidative stress support HIV RNA replication [103]. Maternal HIV infection and ART use can cause foetal immune-metabolic dysregulation [104] which may contribute to impaired growth and long-term abnormal immune development. T-lymphocyte maturation and natural killer (NK) cell numbers or/and functions are altered in HEU new-borns and these abnormalities persist over time [105–107] causing increased mortality and morbidity. This sub-study aims to determine the role of maternal HIV infection on NK cell surface markers and nutrients transporters expression of infants with long-term (> 7 months), medium-term (1–7 months) and short-term (< 1 months) *in utero* ART exposures compared to their HUU counterparts. We will isolate peripheral blood mononuclear cells (PBMCs) for cell staining and analysis. Measurements require samples with sufficient blood volume of ≥2 mL and hence assays will be done later, after 12 months of age. We will determine numbers and function of T-lymphocytes, as well as NK cells as previously described [108]. This longitudinal sub-study will involve convenient sampling of *≥*160 infants at weeks 48 and 96 of age. See Table 3**xxvi** for details.

#### Latent tuberculosis co-infection sub-study (longitudinal analysis)

Co-infection with *Mycobacterium tuberculosis* (MTB) is an independent risk factor for maternal mortality in HIV infection [8]. We hypothesize that undiagnosed latent TB can partially explain increased infant mortality in resource limited settings. Diagnosis of TB can be a clinical challenge. Sputum tests for TB are generally false-negative in a significant fraction of patients due low sensitivity [109]. Tuberculin skin test (TST) has been used to screen for latent TB; however, due to the interaction with BCG vaccine [110] which is administered routinely at birth in Zimbabwe, TST specificity may be greatly reduced in our study population. To test for latent TB, IFN-γ release assays (IGRAs), unaffected by BCG vaccination will be used in a subgroup of HIV- infected and -uninfected women. IGRAs measurements and size of TST lesions will be correlated with adverse maternal and infant outcomes with the aim to develop future more sensitive latent TB diagnostic algorithms for pregnant women in resource limited settings.

This latent TB co-infection sub-study will involve convenient sampling of a total of 400 HIV-infected and-uninfected women. TST will be done in pregnancy and at 6 weeks postpartum with concurrent collection of blood for IGRAs testing. Further, placental samples will be tested for histological lesions suggestive for MTB inflammation. Placenta samples will be randomly collected from *≥*100 mothers at delivery. See Table 3**xxvii** for details.

#### Maternal stress sub-study (longitudinal analysis)

Perinatal stress has been linked to alterations of the hypothalamic-pituitary-adrenal axis activity of the offspring leading to vulnerability later in life. Maternal stress results in increases in cortisol, norepinephrine and inflammation with implications in infant health. Causes of maternal stress include diagnosis of HIV, intimate partner violence, low socio-economic status, being a single parent, unplanned pregnancy, food insecurity including bereavement. This sub-study will assess prevalence, risk factors and coping mechanism of maternal stress among HIV -infected and -uninfected pregnant women. In this maternal stress sub-study, a perceived stress tool will be administered to *≥*400 HIV- infected and - uninfected women in pregnancy and at 6 weeks postpartum.

Stress hormone, cortisol production has a circadian rhythm with levels peaking in the early morning and dropping to the lowest at night, with levels rising independently of circadian rhythm in response to stress [111, 112]. Studies have shown high correlations between serum and salivary cortisol, thus salivary cortisol levels are a reliable estimate of serum cortisol levels [113]. Single salivary cortisol measurement are unreliable as biomarkers for stress since the circadian rhythm of cortisol secretion is not captured [114]. We will therefore measure morning and evening infant saliva cortisol at 6 months of age and correlate levels to child health and their long term mental health and neurocognitive outcomes. Infant cortisol levels will be measured in salivary swabs at week 6 of age. The mothers, following instructions given by the study nurse will collect the infant saliva swabs for cortisol measurements. See Table 3**xxviii** for test details.

#### Neurodevelopmental outcomes (longitudinal analysis)

Neurodevelopmental deficits such as language impairment are more severe and more prevalent in HEI and HEU infants compared with HUU controls in resource limited settings [115]. This sub-study will be evaluating potential long-term neurodevelopmental sequelae related to extended ART exposure. Therefore, a subgroup of HEU, HEI and HUU infants will have extended testing for cognitive performance and neurodevelopmental defects using the Mullen tool [91] at 12 and 24 months correlating with socio-economic status and infections. A total of *≥*300 HEU and HUU infants will be conveniently sampled and followed up.

#### Immunotoxicology sub-study (longitudinal analysis)

Exposure of pregnant women and their infants to mycotoxins is a major public health concern, as this may be associated with nutritional deficiencies and growth retardation. Mycotoxins, especially carcinogenic aflatoxins b1 (AFB1), produced by *Aspergillus* *flavus* are normally present in poorly stored food grains. Not much has been done to determine the maternal burden of mycotoxins, more so in this high HIV prevalence setting. At least in porcine models exposure to mycotoxin, fumonisin B1 has been shown to alter the immune responses by decreasing the level of IL-8 [116].

This sub-study hypothesises that high exposures to mycotoxin causes maternal immune-dysregulation that in turn causes adverse birth outcomes and poor infant health. The main aim is to identity useful biomarkers for interventions to mitigate mycotoxin exposure associated adverse birth outcomes and poor health in infants. Maternal random urine, amniotic fluid and longitudinal breast milk samples will be analysed for at least 20 mycotoxin biomarkers, including both the parent mycotoxins and mycotoxin metabolites. High-performance liquid chromatography tandem mass spectrometry method with modifications will be used as previously described [117]. A total of *≥*400 HIV- infected and -uninfected women will be randomly selected in pregnancy, and followed up at delivery and 24 months after delivery.

#### *Helicobacter pylori* sub-study (longitudinal analysis)

The development of chronic gastritis, recurrent peptic ulcers including gastric cancers has been associated with *H. pylori* infections [6]. Risk factors include low socio-economic status, poor hygiene, and overcrowding [118]. The prevalence of *H. pylori* infection is persistently higher in developing countries where more than 70% of the adult population is colonized with *H. pylori,* with > 50% children acquiring it by the age of 10 [119]. Most published data on *H. pylori* infection are largely centred on adults. Thus, there are significant knowledge gaps on the risk factors of initial *H. pylori* colonization, trends and persistence of the infection in children. This sub-study aims to compare timing and trends of *H. pylori* colonisation and immunity among HUU and HEU infants from birth until 2 years of age, with the aim to develop preventive measures to reduce early life infections. Random sampling of ≥100 women in pregnancy will be done. Follow up will be within 10 days of life, at weeks 10, 14, 24, 36, 48, 72 and 96 with collection of MIPs stool samples for testing of *H. pylori* antigen and antibodies using simple ELISAs. See Table 3**xxix** for details.

### Study time frame

Pregnant women have been enrolled into the UZ-CHS Birth Cohort study from February 2016 until June 2019. MIPs will be followed for 2 years, with an expected study completion date in June 2021. Long-term follow-up studies until school-going age and adolescence are in planning.

### Data flow and analysis

Data is being entered at the study sites into paper CRF that will subsequently be entered into a Research Electronic Data Capture (REDCap) secure web with regular backups. Data will be analysed using SPSS (version 17.0, Chicago, IL) or other analysis tools using standard statistical methods for parametric or non-parametric testing or analysis of categorical outcomes, whatever appropriate.

The main endpoint of our study, the composite endpoint of stillbirth and mortality until two years of age of HEU vs. HUU infants will be corrected for confounders in a multivariable logistic regression analysis. In this analysis, we aim to correct for socio-demographic parameters (e.g. maternal age), descriptors of the current and past reproductive history (e.g. number previous pregnancies, current twin/multiple pregnancy), maternal comorbidities (e.g. hypertension, metabolic syndrome, infectious comorbidities), economic situation (marriage and employment state, family income), maternal nutrition (e.g. number days without food, MUAC) and hygiene (number of days without running water). For selection of confounders, individual parameters from the database will be tested in a univariate analysis. If significance is detected (*p* < 0.1), the parameter will be included in a multivariable analysis with parameter elimination for optimization of the Akaike information criterion. Confounders for maternal outcomes will be selected using a similar strategy.

For secondary outcomes, Cox proportional hazard models will be used for time-dependent variables, also controlled for confounders in multivariable analyses. Predictors of secondary outcomes will also be analysed using hierarchical all-against-all clustering [120]. A *p*-value < 0.05 will be considered significant. We will provide nominal *p*-values and Bonferroni-corrected *p*-values in case of a multiple testing situation.

## Discussion

The UZ-CHS Birth cohort is a prospective study in a resource limited setting following up HEU and HUU infants, assessing their health and development within the first 2 years of life. The composite primary endpoint is stillbirth and infant mortality within the first two years of life in HEU versus HUU children. Maternal mortality from enrolment until two years after birth in HIV infected vs. HIV uninfected participants is another primary endpoint of this study. Secondary endpoints include a range of maternal, birth and infant outcomes. It provides a unique opportunity to answer a multitude of research questions that build a comprehensive understanding of the impact of HIV and comorbidities, ART use including other maternal and infant environmental factors on infant mortality, morbidity, growth and immune/ neurodevelopment. The potential output will impact on policy and practice regarding perinatal care for mothers and infants in resource limited settings. The extensive data on maternal nutrition and hygiene, sexual and reproductive behaviour, HIV disease characteristics, infectious disease complications and the extensive bio-sampling of MIPs provide opportunities for a number of sub-studies to better understand how environmental exposures modulate immune system, microbiota and other physiological parameters in mothers and their infants.

Health of HEU infants is of great concern and great public health importance in SSA; however, the reason for poor outcomes in HEU is not well understood. Besides exposure to HIV and ART, HEU infants might differ from HUU by exposure to other infections and maternal co-morbidities and other socio-economic measures might also differ. For these reasons, a comprehensive study is needed. Results of our analyses might identify early markers for poor outcome and help to develop specific interventions for HEU infants. For instance, if the response of HEU and HUU infants to vaccines would differ, the vaccination plan for HEU infants could be modified [121]. On the other hand, the possibility that exposure to HIV *in utero *would prime the immune system with lasting effects after birth warrants further studying and could yield in insights regarding immune development.

**Fig. 1:**
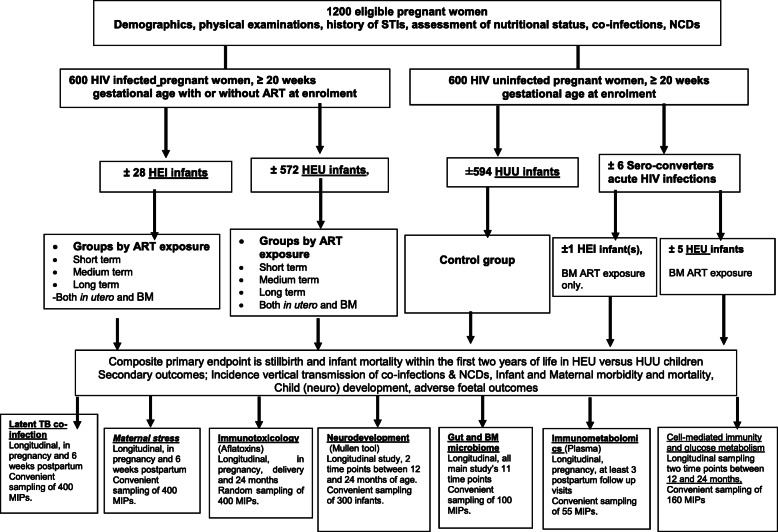
Main Study and sub-studies Flow Diagram. ART: Antiretroviral therapy, BM: Breast milk, Co-infections include hepatitis B virus, hepatitis C virus, CMV:cytomegalovirus, and intestinal parasites. HEI: HIV exposed and infected, HEU: HIV exposed but uninfected HIV: Human immunodeficiency virus, HUU: HIV unexposed and uninfected, MIPs: Mother -infant pairs, NCD: Non-communicable diseases and these include hypertensive disorders, malnutrition and diabetes, STIs: Sexually transmitted infections

However, Option B+ strategy does not completely eliminate MTCT and among the offspring of 600 HIV- infected mothers, approximately 30 HEI infants are expected (Fig. 1). Mortality of these infants is expected to be significantly higher compared to HEU infants, warranting separate analyses of this group for all clinical analyses and biomarker assays.

Introduction of ART has dramatically decreased the MTCT rates by more than 10-fold, an important medical success [2, 122, 123]. However, use of ART in pregnancy is a double edged sword and part of the burden on HEU infants might be due to adverse effects of ART. The safest ART regimens in pregnancy still needs to be determined. ART treatment plans continue to evolve and plans to replace Efavirenz with Dolutegravir are at an advanced stage but with its challenges [53]. Efavirenz has been shown to trigger apoptosis, an effect in part due to interference with mitochondrial membrane potential [48]. Continuous surveillance regarding Dolutegravir related structural birth defects can be done in future sub-studies in our cohort.

Future sub-studies of our cohort might follow infants into childhood and adolescence to assess long term immune and neurodevelopment including mental health outcomes. A further desirable future expansion might be a strictly rural observational cohort, to control for household, lifestyle and environmental confounders associated with the current urban based study.

Strength of our study includes the large number of pregnant women as well as extensive longitudinal questionnaires addressing many aspects of health, nutrition, hygiene and reproductive behaviour. Bio-sampling of many biomaterials at close time points from both, mothers and infants, will enable identification of biomarkers and addressing of pathomechanisms in future translational experiments. In our study, we will document timing of ART before, during and after pregnancy in detail, allowing studying the effects of foetal exposure to ART at different time points on birth outcomes and infant developments. Furthermore, our study has a comparative group of HUU infants enrolled from the same community sampled at the same time. Finally, even though Zimbabwe currently faces pronounced economic challenges, literacy is nearly universal. Research participants should understand study procedures well, and mothers will be able to document infant health in morbidity diaries and follow the study processes with high compliance.

Limitations of our study include the non-interventional study design. Therefore, any important association identified would need to be confirmed by an interventional study. Further, gestation age estimate is based on reported last menstrual period that may be associated with recall bias and complementary use of ultrasound is missing. Moreover, due to the high prevalence of environmental risk factors for infant health related to poor socio-economic status, some specific risk factors might not be identified by our study design. Therefore, there is the possibility of a type II error for adverse effects of ART with low risk in our cohort due to a high number of additional risk factors. Finally, all our research participants reside in a rather homogeneous high-density residential area in Harare, Zimbabwe. Our results will therefore probably apply to similar resource limited settings in SSA. However, results from our cohort might not be generalizable to other more affluent urban environments or rural areas. Caution would be needed to generalize our results to industrialized countries.

In summary, we introduce the University of Zimbabwe birth cohort study, which is following up 600 HIV infected and 600 uninfected mothers and their infants in a resource limited setting in a high-density residential areas around Harare. Acquisition of extensive clinical meta-data and comprehensive bio-sampling will enable us to answer many questions important for this study population including the health of HEU infants. This is important, as future health and wellbeing in adulthood are influenced by external environmental insults encountered during the first couple years of life.

## Data Availability

The datasets obtained during this study will be available upon request to the corresponding authors. Only encrypted data will be shared with collaborating partners using EGAcryptor in line with the current European General Data Protection Regulation effective May 25, 2018. Results will be published in journals with scientific quality assurance, targeting open access journals whenever possible for a wider accessibility. Data sets used in published studies will be made publicly available with the respective publication.

## Abbreviations

AIDS: Acquired immunodeficiency syndrome
ART: Antiretroviral therapy
CD4: Cluster of differentiation 4
CD8: Cluster of differentiation 8
CMV: Cytomegalovirus
DBS: Dried blood spots
DNA: Deoxyribonucleic acid
EPI: Expanded program on immunization
JREC: Joint Research Ethics Committee of the University of Zimbabwe and Parirenyatwa Hospital
HAZ: Height for age Z score
HCAZ: Head circumference for age Z score
HBV: Hepatitis B virus
HEU: HIV-exposed but uninfected
HEI: HIV exposed and infected
HIV: Human immunodeficiency virus
HBsAg: Hepatitis B virus surface antigen
HUU: HIV-unexposed and uninfected
LBW: Low birth weight
LMP: Last menstrual period
IL: Interleukin
MIPs: Mother-infant pairs
MTCT: Mother to child transmission
MUAC: Mid upper mid arm circumference
MRCZ: Medical Research Council of Zimbabwe
NCD: Non-communicable diseases
PCR: Polymerase chain reaction
PMTCT: Prevention of mother to child transmission of HIV
ZHDS: Zimbabwe health demographic survey
RNA: Ribonucleic acid
SD: Standard deviation
SFH: Symphysis fundal height
SGA: Small for gestation age
SSA: Sub Saharan Africa
TB: Tuberculosis
TENOLAM-E: Tenofovir, Lamivudine and Efavirenz
USA: United States of America
UZ-CHS: University of Zimbabwe College of Health Sciences
WAZ: Weight for age Z scores
WHZ: Weight for height Z score
WHO: The World Health Organisation
